# A high-resolution spatiotemporal morphological dataset: Port Aransas beach, Texas

**DOI:** 10.1016/j.dib.2024.110948

**Published:** 2024-09-14

**Authors:** Marina Vicens-Miquel, Philippe Tissot, Deidre D. Williams, Katherine F.A. Colburn, Matthew Kastl, Savannah Stephenson

**Affiliations:** a Texas A&M University-Corpus Christi: Conrad Blucher Institute, Corpus Christi, TX 78412, USA; b NSF AI Institute for Research on Trustworthy AI in Weather, Climate, and Coastal Oceanography (AI2ES), Norman, OK 73019, USA

**Keywords:** Beach morphology, AI-ready data, Coastal inundation, Topographic survey, Beach profiles, Coastal imagery, Mustang Island, TX

## Abstract

The study of beach morphology holds significant importance in coastal management, offering insights into coastal and environmental processes. It involves analyzing physical characteristics and beach features such as profile shape, slope, sediment composition, and grain size, as well as changes in elevation due to both erosion and accretion over time. Furthermore, studying changes in beach morphology is essential in predicting and monitoring coastal inundation events, especially in the context of rising sea levels and subsidence in some areas. However, having access to high-frequency oblique imagery and beach elevation datasets to document and confirm coastal forcing events and understand their impact on beach morphology is a notable challenge. This paper describes a one-year dataset comprising bi-monthly topographic surveys and imagery collected daily at 30 min increments at the beach adjacent to Horace Caldwell Pier in Port Aransas, Texas. The data collection started in February 2023 and ended in January 2024. The dataset includes 18 topographic surveys, 6879 beach images, and ocean/wave videos that can be combined with colocated National Oceanic and Atmospheric Administration metocean measurements. The one-year temporal span of the dataset allows for the observation and analysis of seasonal variations, contributing to a deeper understanding of coastal dynamics in the study area. Furthermore, a study that combines survey measurements with camera imagery is rare and provides valuable information on conditions before, after, and between surveys and periods of inundation. The imagery enables monitoring of inundation events, while the topographic surveys facilitate the analysis of their impact on beach morphology, including beach erosion and accretion. Various products, including beach profiles, contours, slope maps, triangular irregular networks, and digital elevation models, were derived from the topographic dataset, allowing in depth analysis of beach morphology. Additionally, the dataset contains a time series of four wet/dry shoreline delineations per day and their corresponding elevation extracted by combining the imagery with the digital elevation models. Thus, this paper provides a high-frequency morphological dataset and a machine learning-ready dataset suitable for predicting coastal inundation.

Specifications Table

**Table:** 

Subject	Earth and Planetary Science
Specific subject area	Beach Morphology, Coastal Inundation, and Applied Machine Learning
Data format	1-year time series of (1) bi-monthly topographic survey data, beach profiles, contours, slope maps, triangular irregular networks (TINs), and digital elevation models (DEMs); (2) 30 min oblique imagery during daylight; and (3) a time series of four wet/dry shorelines delineations per day and their corresponding elevation.
Type of data	(1) Topographic survey data (raw data), beach profiles, elevation contours, and the wet/dry shorelines delineations are geodatabases feature classes; (2) slope maps and DEMs are raster datasets; (3) TINs are provided as TIN datasets; (4) imagery is provided as tiff files; (5) graphs are provided as jpeg files; and (6) the wet/dry shoreline elevation is provided as a CSV file.
Data collection	The data were collected, and the products were derived by the Coastal Dynamics Lab team at the Conrad Blucher Institute for Surveying and Science (CBI), Texas A&M University-Corpus Christi (TAMU-CC). This included (1) topographic survey data; (2) survey products such as elevation contours, slope maps, TINs, and DEMs; (3) imagery collected from a set of cameras installed and maintained on pier at the study site; (4) graphics representing the change of sand volume between surveys generated from the survey data; and (5) wet/dry shoreline delineation and wet/dry shoreline elevation.
Data source location	The suite of data was collected along a relatively small (54.9 × 85.3 m) beach-protected segment along the Gulf-facing beach adjacent to the Horace Caldwell Pier in Port Aransas, (27°49′40.5" N, 97°03′10.8" W) on Mustang Island, Texas.
Data accessibility	Amazon Web Service (AWS): http://port-aransas-morphological-beach-data.s3-website-us-east-1.amazonaws.com/ Data Identification Number: 10.5281/zenodo.12746270

## 1 Value of the Data

• The dataset described in this article can be utilized (1) to analyze and understand changes in beach morphology along the beach adjacent to Horace Caldwell Pier, Mustang Island, Texas; (2) to monitor coastal inundation events and understand their impacts on the beach morphology; (3) to gain insight into seasonal patterns in beach morphology changes and inundation events; (4) as a machine learning-ready dataset for predicting coastal inundation.
• The most significant contribution of this paper is the creation of a one-year high-frequency dataset encompassing bi-monthly topographic surveys, 30 min imagery, and four daily wet/dry shoreline delineations with their corresponding elevations. This dataset holds significant value for studying beach morphology and predicting coastal inundation events.
• Ocean and wave videos collected by the author are shared via WebCOOS [1]. There is also colocated water level, wind, and wave data accessible via NOAA [2]. The ability to combine the author's data with other colocated metocean data increases its value, as they complement each other well.
• The dataset will be valuable for a diverse audience, including coastal and environmental managers and researchers, data scientists, and others interested in the study of beach morphology and/or coastal inundation events.

## 2 Background

The motivation behind compiling this dataset stems from the need to understand the complex, highly non-linear processes driving changes in beach morphology and coastal inundation due to storm events. Observing and quantifying such changes in real-world beach environments is challenging due to the typically harsh and energetic conditions. However, these measurements are essential for studying these processes, especially as relative sea level rise increases inundation frequencies and coastal erosion. This dataset is particularly valuable as it combines co-located measurements, including camera observations, high-frequency ground surveys, tide gauge data, and wind measurements. Such comprehensive data allow for a thorough analysis of total water levels, comparisons with average water levels, and studies on beach erosion. The total water levels are defined as the combination of tides, surge, and wave runup [3], while average water levels are derived from the 6 min NOAA tide gauge readings, which represent the average of 181 1 Hz measurements taken every 6 min [4]. This dataset has been utilized in two articles published in the Journal of Coastal Research [5,6], providing a more holistic understanding of coastal dynamics and erosion processes. This data article adds value to the original research by offering a robust and detailed dataset that supports in-depth analyses of the coastal dynamics and enhances the reproducibility and validity of the findings presented in the research articles.

## 3 Data Description

The dataset presented in this article captures the morphological variability along a small beach segment on Mustang Island that is located adjacent and north of Horace Caldwell Pier in Port Aransas, Texas. Understanding the morphological changes that occur at beaches requires detailed data from the study area. While many studies, such as [7,8], focus on long-term changes in beach morphology and contribute significantly to long-term planning and beach conservation efforts, our research emphasized the creation of a high spatiotemporal frequency dataset. This high-frequency dataset includes digital imagery collected multiple times per day that captures, detailed and rapid changes in the total water levels. For the survey data, high frequency refers to bimonthly surveys, which are considered very frequent for this type of dataset collection. Bimonthly surveys are rare in beach morphology studies, as they offer a unique temporal resolution that allows for the assessment of morphological changes over shorter timescales, used in concert with the suite of multiple daily observations interpreted from the digital imagery.

Unlike traditional datasets that typically provide lower frequency observations, this dataset uniquely enables the assessment of the immediate impacts of metocean forcings, such as waves and water levels, on beach morphology. It allows for the analysis of both rapid, short-term changes and more gradual alterations in the beach profile, offering insights that are not possible with less frequent data collection methods. This high-resolution data can be particularly valuable for understanding dynamic coastal processes, informing short-term management decisions, and enhancing predictive models for coastal erosion and beach morphology changes.

All the survey data and derived products are presented in meters in accordance with SI units. The timestamps on the imagery are in UTC to avoid complications due to daylight-saving time changes.

## 4 Experimental Design, Materials and Methods

### 4.1 Study Area – Horace Caldwell Pier, Port Aransas, Texas

**Fig. 1:**
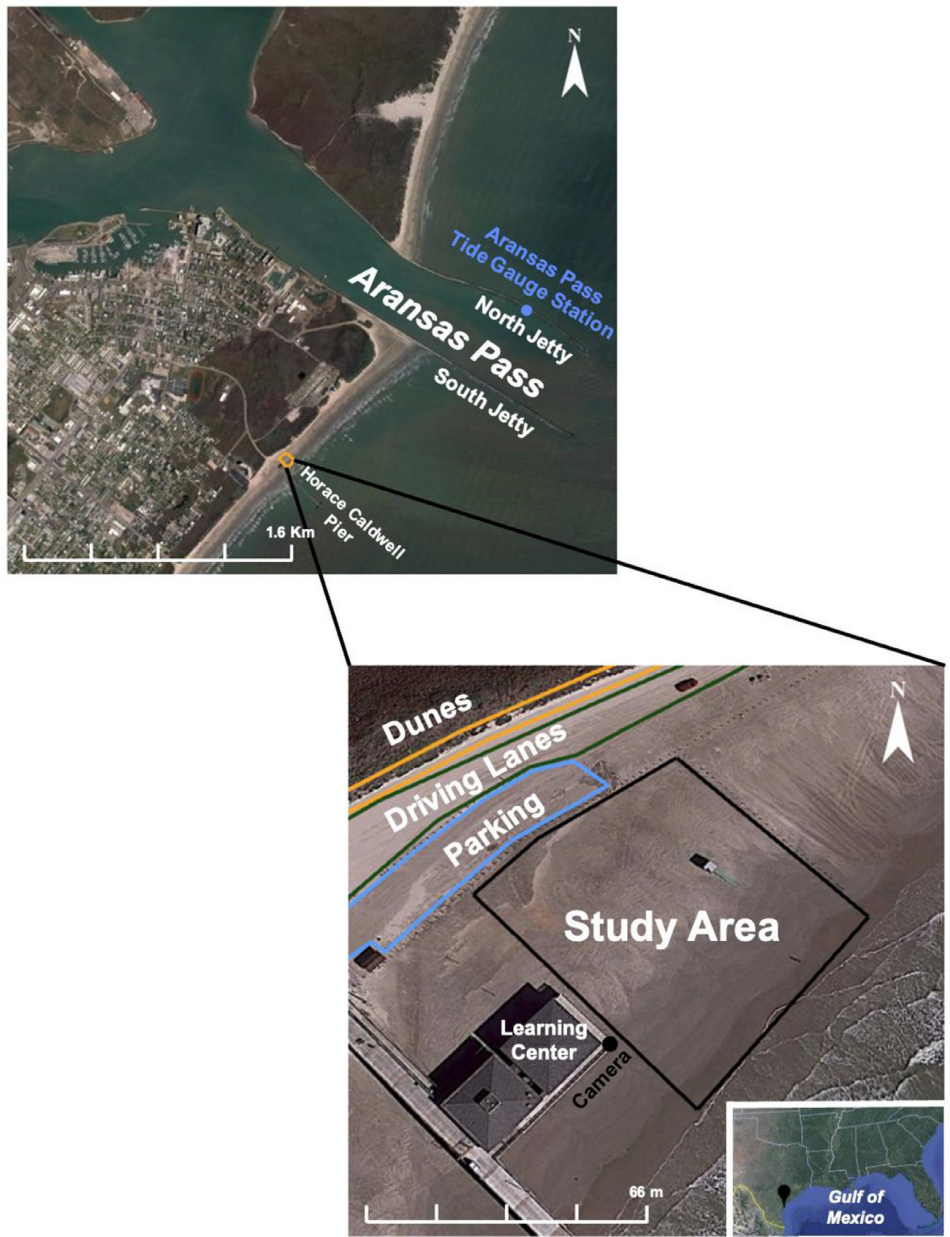
Illustration of the study area, highlighting the dunes, driving lanes, pier, jetties, and the specific beach segment studied.

The dataset encompasses measurements from the beach segment located north of and adjacent to Horace Caldwell Pier in Port Aransas, Texas. The study area represents a small beach segment measuring 54.9 × 85.3 m located on the 29-km long Mustang Island, part of the central Texas coastline often referred to as the Coastal Bend (refer to Fig. 1). The beach morphology in the study area exhibits unique characteristics influenced by both natural and anthropogenic factors. Some of these factors include the impact of coastal structures such as piers and jetties, restricted vehicular traffic along an otherwise high-use beach, and a microtidal range. The pier affects wave shadowing and refraction dynamics, while the jetty influences longshore sediment transport. Additionally, Texas is characterized by a microtidal range, meaning metocean forces have a greater impact on water levels and inundation compared to areas with a larger tidal range. A significant feature of the study area is its restriction on vehicular traffic. Vehicles are allowed on most Texas beaches [9], causing significant challenges in studying temporal changes in beach morphology due to sand redistribution both by the vehicles but also due to maintenance of the beach driving lanes. Consequently, the study area near Horace Caldwell Pier offers a more stable and less altered environment for research compared to other Texas beaches. Furthermore, several factors contributed to the selection of this study area, including (1) the proximity to the pier was essential for camera installation and power access; (2) the restricted access to the pier provides security to prevent vandalism and ensure uninterrupted data collection; (3) vehicular traffic limited to only lifeguards, thereby minimizing the daily anthropogenic impact on beach morphology.

### 4.2 Topographic Survey Data Acquisition Process

**Fig. 2:**
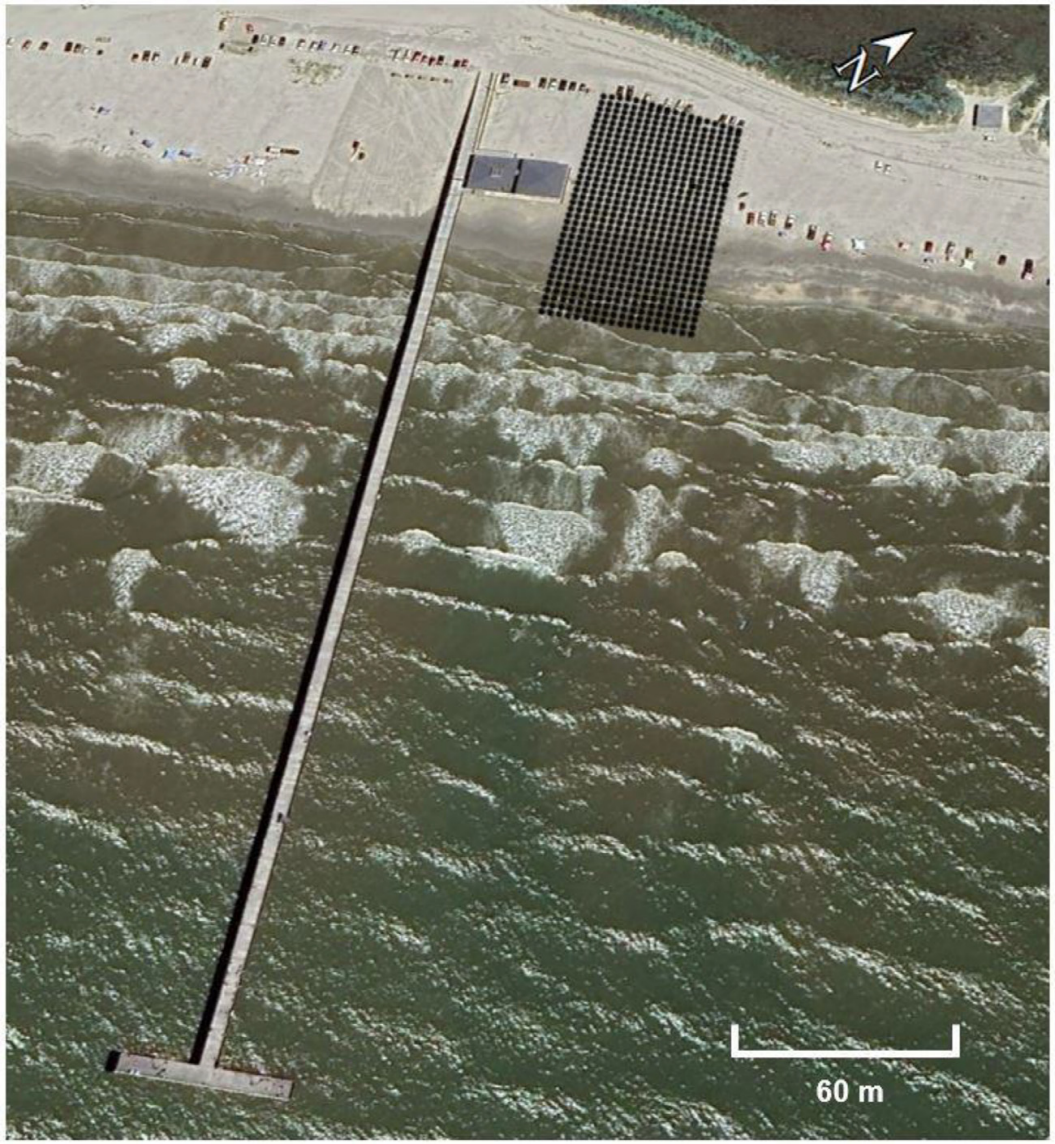
Illustration of the 3.05-meter (10 by 10 US ft) topographic survey grid used during the data collection process.

All survey data was collected by the authors and their team. The topographic surveys utilized Real-Time Kinematic (RTK) positioning with a Trimble R10 Global Navigation Satellite System (GNSS) multi-frequency receiver, receiving broadcast corrections from Trimble Virtual Reference Stations (VRS) using the G3 CMRx NAD83 2011 model (Trimble, accessed in 2024). Trimble reports horizontal and vertical accuracies (RMS) of 2 and 3 centimeters, respectively. The x and y coordinates were referenced to NAD 1983 State Plane Texas South, while elevation measurements were based on NAVD88. Although all surveys were conducted using a 3.05 by 3.05-m grid (10 by 10 US ft) (refer to Fig. 2), the lengths of the surveys varied. This variation was due to the surveys being performed up to the waterline, with the waterline's position at the time of the survey determining the survey length.

### 4.3 Imagery Acquisition Process

**Fig. 3:**
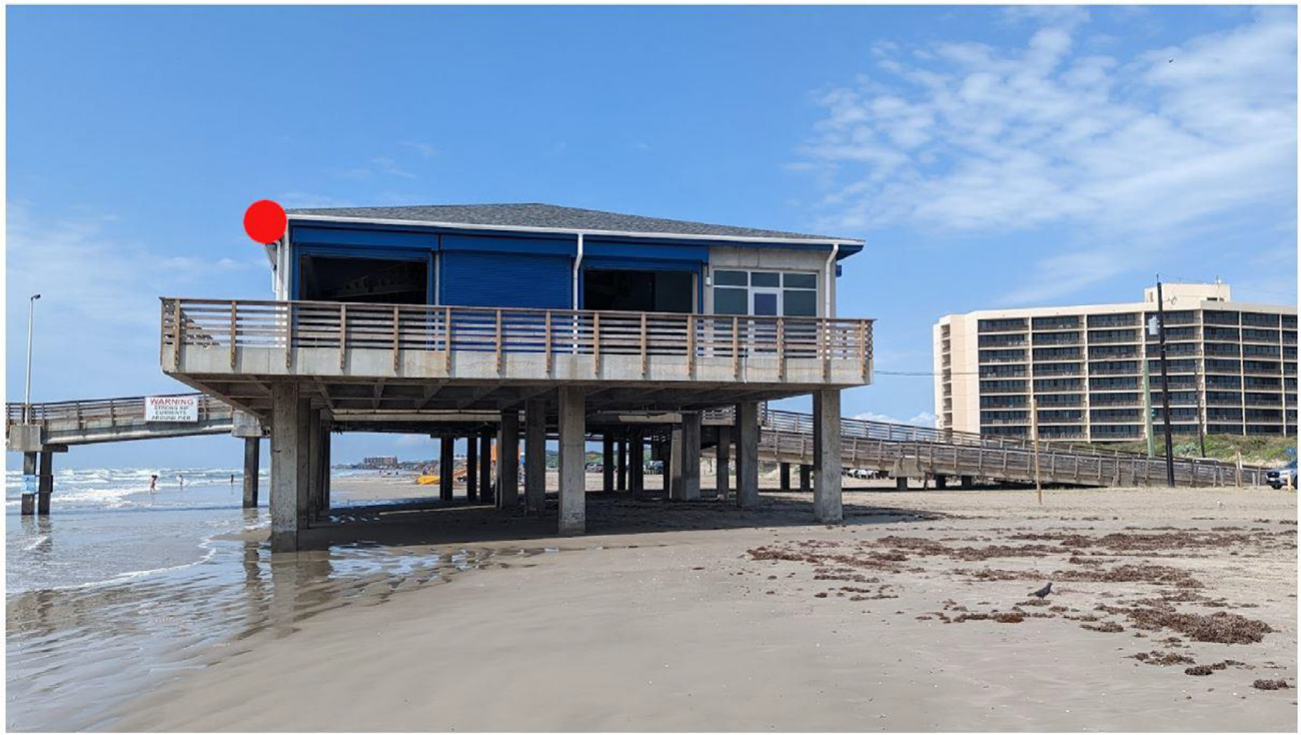
Illustration showing a section of the pier and the Horace Caldwell Pier Learning Center building, where the camera and all associated equipment and computers were installed. The red dot indicates the location of the cameras.

Collecting imagery of the study area is essential for monitoring the frequency and intensity (landward extent and period) of inundation events. These data were correlated with metocean variables and changes in beach morphology. To support this analysis, a camera was installed to capture oblique photographs every 30 min. The camera equipment was installed at the Horace Caldwell Pier Learning Center building (refer to Fig. 3), which facilitated access to power, provided an elevated viewpoint for the cameras, and provided housing for the associated computer and networking equipment. The protected, restricted-access location, also reduced the likelihood of vandalism and damage during storms and other extreme events. The installation involved two protective boxes with desiccant to shield the equipment from salt and sand and reduce lens fogging, which could damage the equipment and affect image quality.

One box, located inside the building, houses an on-site Zed Box Nvidia Jetson computer [10], which runs a Python program to capture imagery every 30 min and upload the data to Amazon Web Services (AWS) [11]. This box also contains a Sierra Wireless AirLink cellular router [12] for internet connectivity. Additionally, a Power over Ethernet (PoE) [13] cable connects the camera to the Jetson computer. To ensure data security, a hard drive [14] is included in this setup to back up all data locally in addition to the uploads to AWS.

The camera responsible for the oblique images, an Amcrest 4K Outdoor Security IP Turret PoE Camera (IP67 IP8M-T2599EW) [15], is located in the outside box. This 8-megapixel camera has a wide 125-degree viewing angle. An additional identical camera was added to the outside box to monitor the ocean and waves. This camera is live and can be accessed in real-time and also past stored videos though WebCOOS [1]. The data collection method developed is designed to be replicable by other researchers. Consequently, the total cost of the setup incurred in 2022 was under $2,000, with the possibility of reducing it to $750 by replacing the Jetson computer with a Raspberry Pi.

### 4.4 Data Organization

The data is organized and structured as follows:

• Geodatabase
  ○ Topographic survey data (CSV)
  ○ DEMs (Digital Elevation Models)
  ○ Contour profiles
  ○ Slope maps
• TINs (Triangulated Irregular Networks)
• Beach Profiles Plots
  ○ Individual beach profile plots (pdf)
  ○ Comparison of consecutive beach profile plots (pdf)
• Imagery (TIFF)
  ○ Wet/Dry Shoreline Elevation
  ○ Wet/dry shoreline elevation time series (CSV)
  ○ Figure comparing the wet/dry shoreline elevation with colocated tide gauge water levels observations and harmonic predictions

The geodatabases are ready for import into any Geographic Information System (GIS) software. Each geodatabase corresponds to a single survey, resulting in two geodatabases per month due to the bi-monthly survey frequency. Each survey contains topographic survey data collected on a 3.05 by 3.05 m (10 by 10 US ft) grid. DEMs, contour profiles, and slope maps are derived from the topographic survey data. The TINs are stored in a separate folder rather than within a geodatabase. However, they are also easy to import into GIS software and are created from the original topographic dataset.

The beach profile plots folder contains two PDF files. The first one contains individual beach profile plots for each survey. The second includes comparisons of consecutive beach profiles, providing valuable information on the short-term dynamics of beach morphology.

The imagery folder contains all images collected between 8 AM and 5 PM CDT, ensuring consistent lighting conditions. The timestamps on the imagery file names are in UTC, while the timestamps in the top left corner of the images are in local time (CDT).

The wet/dry shoreline elevation folder contains a CSV file with wet/dry shoreline elevations extracted from the imagery overlaid on the DEM models. This time series of elevations includes four measurements per day. Additionally, the folder includes a plot comparing the wet/dry shoreline elevation with colocated tide gauge water level observations and harmonic predictions.

### 4.5 Illustrative Data

This section presents sample datasets to facilitate data interpretation. Since survey data and their products are common and widely used in similar studies, they will not be shown here. Instead, this section will describe only the imagery, beach profiles, and total water level time series datasets.

**Fig. 4:**
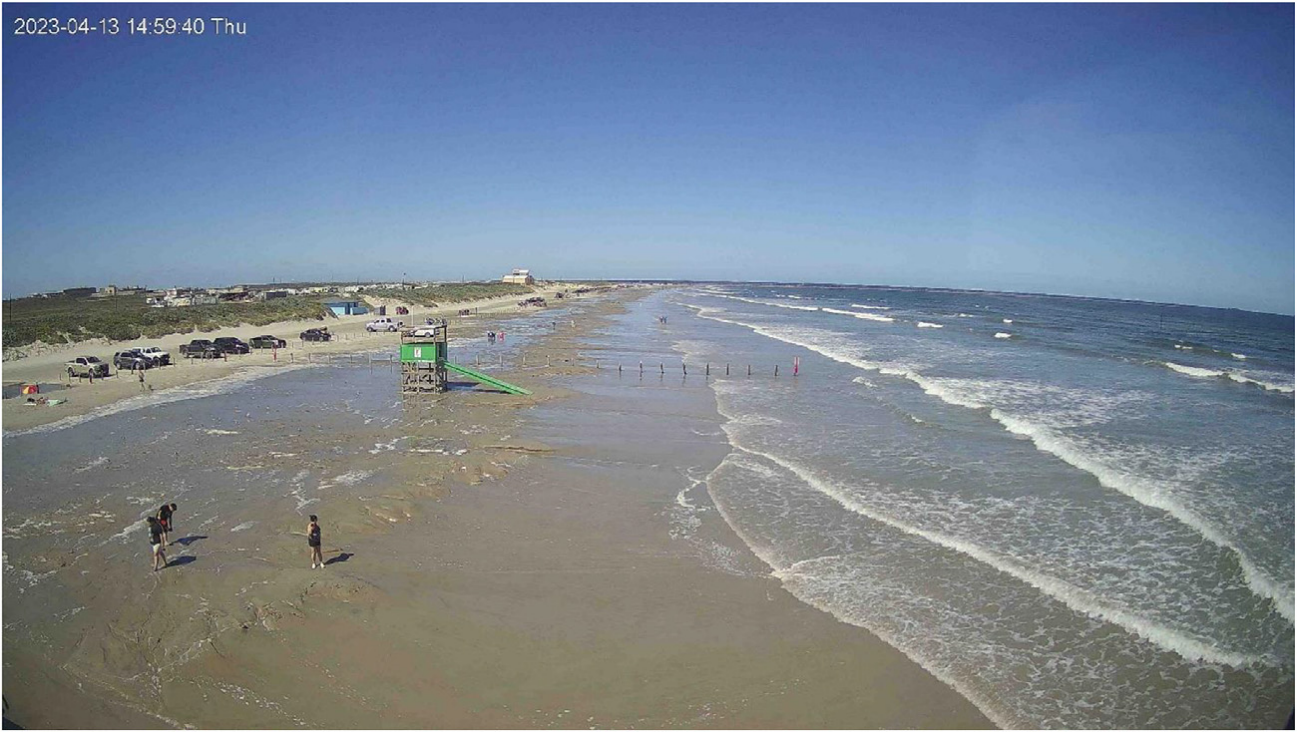
Illustration of an inundation event captured on April 13th, 2023, at 20:59 pm UTC (14:59 pm CDT), showcasing the impact of an inundation event on the study area, demonstrating the landward extent of inundation.

Fig. 4 illustrates a sample image collected by the camera. On the left side of the image, the dunes are visible; in the middle, the bollards that demarcate our study area can be seen; and on the right, the landward extent of the water is observable, as well as the presence and type of waves. The image below shows an inundation event on April 13th, 2023, highlighting the importance of having cameras to monitor such events. Imagery is essential for understanding beach conditions and events, as it provides a visual record of these occurrences. This visual record complements and enhances the interpretation of changes in beach morphology that are reflected in the beach profile survey data. Without these images, interpreting the changes in beach morphology relative to inundation would be significantly more challenging. For this study area, [5] defined two types of inundation events: full beach inundation and partial beach inundation. A full beach inundation event is characterized by water covering the beach up to the bollards near the driving lane and back dunes, while a partial beach inundation event occurs when water reaches up to the middle of the berm without extending to the bollards.

**Fig. 5:**
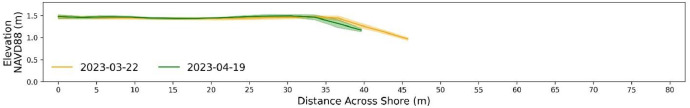
Comparison of beach profiles illustrating changes between two consecutive surveys.

Beach profiles are extensively referred to in literature describing beach morphology [16,17]. Profile data describe the physical characteristics of the beach and its features from the landward limit to the water's edge. Beach profiles provide a detailed cross-sectional view of the beach terrain, capturing variations in elevation, slope, and accretionary features along a transect perpendicular to the shoreline [18,19]. Fig. 5 illustrates a comparison between two consecutively surveyed beach profiles. The darker color represents the mean beach profile, computed by averaging all alongshore measurements. The lighter color band (ribbon) indicates the variability in these measurements, computed as two standard errors of the mean alongshore survey measurements.

The comparison in Fig. 5 is particularly valuable as it highlights the impact of an inundation event (Fig. 4) on beach morphology. By comparing the beach profiles before and after the hurricane, we can discern its effects, such as foreshore erosion and landward sand redistribution.

One of the primary objectives was to create a dataset of total water levels specifically designed for use in machine learning models that are applied to predict coastal inundation. This `AI-ready' dataset is fully prepared for direct use as input in machine learning applications, eliminating the need for additional preprocessing steps. Predicting coastal inundation is increasingly crucial due to ongoing sea level rise and subsidence, which continuously increases the frequency and severity of these events. The total water level is measured as the elevation of the wet/dry shoreline resulting from a combination of tides, surge, and wave runup [20].

**Fig. 6:**
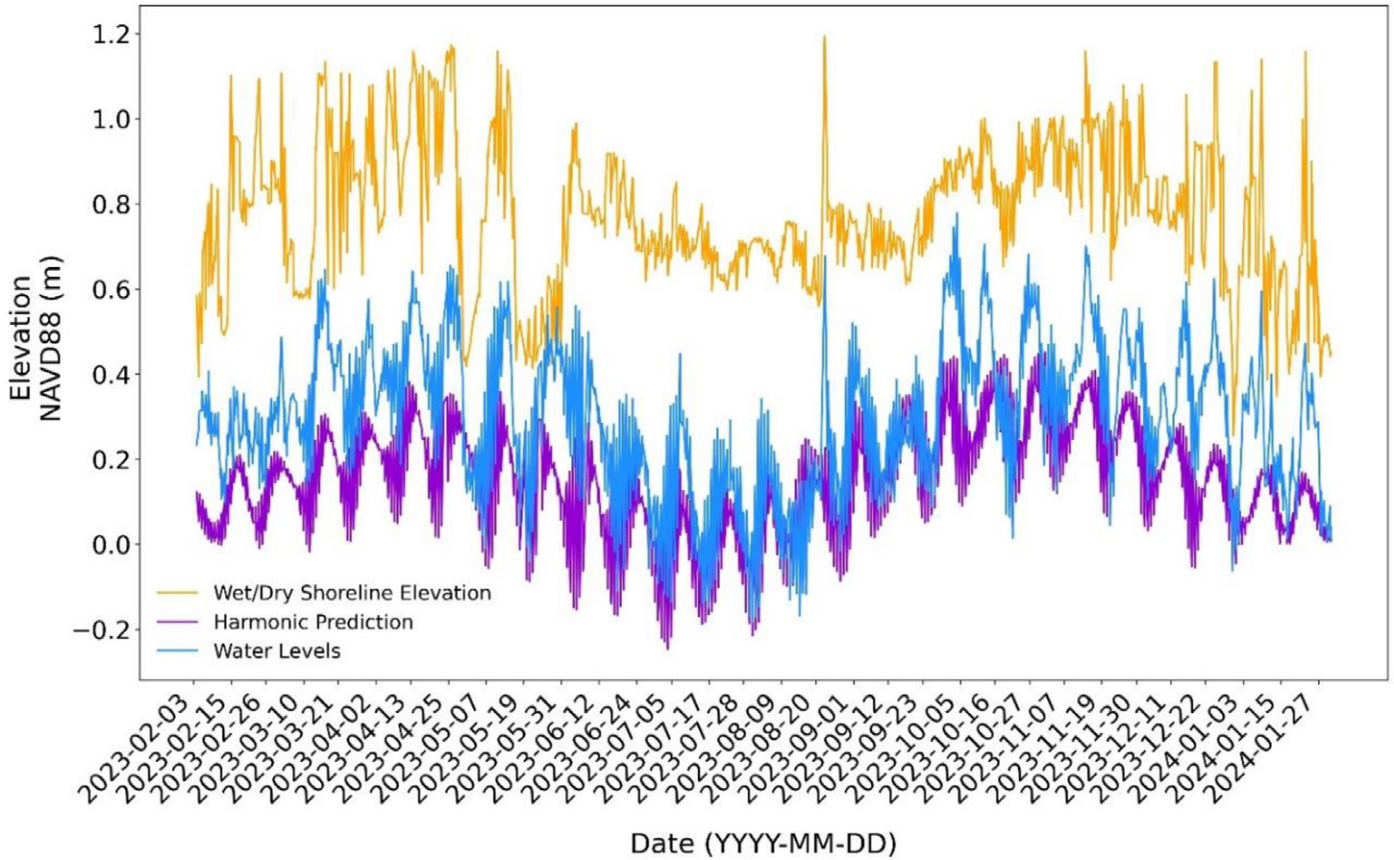
Comparison of total water level, observed tide gauge water level, and the harmonic prediction time series for the study area and the nearby Port Aransas tide gauge station.

Fig. 6 compares the time series of wet/dry shoreline elevations with water level and harmonic predictions. This figure highlights the need and significance of having a consistent time series of total water levels, which typically measure approximately 0.5 meters higher than the water level readings for the study area. This disparity is even more pronounced when compared to harmonic predictions, which are often inadequate for accurately forecasting water levels at tide gauges along the Texas coast. This dataset provides an opportunity to compare and study colocated water levels measured at a tide gauge, tidal predictions, and total water levels, which are the more relevant time series for coastal inundation studies.

## Limitations

The main limitation of the dataset is that it only covers a small beach segment of 54.9 × 85.3 m. However, the high frequency of survey measurements makes it a unique and valuable dataset that can be applied to understanding the dynamics of nearby wide isolated beach segments in the Port Aransas area and possibly near Packery Channel. A second limitation is that although the imagery is captured every 30 min, the cameras are not equipped for night-time visibility. Consequently, the dataset includes only daytime imagery. The third limitation is that the surveys and all derived products have varying lengths and, in particular, seaward limits because they did not extend beyond the waterline. A third limitation is that while the 1-year span of the data captures the seasonal variability, it is still a relatively short timespan to study dynamic changes in beach morphology. Finally, there is also a small inherent error in RTK observations, with horizontal and vertical accuracies of 2 and 3 centimeters, respectively, which is common in all RTK-based measurements in beach environments. To account for uncertainties in the wet/dry shoreline position and its elevation, a 30 cm horizontal buffer around the line was included, and 5 % of the measurements representing the maximum and minimum were removed when computing the total water level to eliminate possible outliers and enhance data accuracy.

## Ethics Statement

The current work meets the ethical requirements for publication in Data in Brief and does not involve human subjects, animal experiments, or any data collected from social media platforms.

## CRediT authorship contribution statement

**Marina Vicens-Miquel:** Conceptualization, Methodology, Writing – review & editing. **Philippe Tissot:** Supervision, Conceptualization, Methodology, Writing – review & editing. **Deidre D. Williams:** Methodology, Writing – review & editing. **Katherine F.A. Colburn:** Methodology, Data curation. **Matthew Kastl:** Methodology, Data curation. **Savannah Stephenson:** Methodology, Data curation.

## Data Availability

Port Aransas Morphological Beach Data (Original data) (Zenodo).
